# Six-monthly appointment spacing for clinical visits as a model for retention in HIV Care in Conakry-Guinea: a cohort study

**DOI:** 10.1186/s12879-017-2826-6

**Published:** 2017-12-13

**Authors:** Cavin Epie Bekolo, Abdourahimi Diallo, Mit Philips, Joseph-Desire Yuma, Letizia Di Stefano, Stéphanie Drèze, Jerome Mouton, Youssouf Koita, Ousseni W. Tiomtore

**Affiliations:** 1 Médecins Sans Frontières, Conakry, Guinea; 2 Médecins Sans Frontières, Brussels, Belgium; 3 National HIV/AIDS/STI Treatment & Prevention Programme, Conakry, Guinea; 4 Médecins Sans Frontières, Southern Africa Medical Unit (SAMU), Cape Town, South Africa

**Keywords:** ART delivery, Retention in HIV care, Ebola epidemic, Guinea

## Abstract

**Background:**

The outbreak of the Ebola virus disease (EVD) in 2014 led to massive dropouts in HIV care in Guinea. Meanwhile, Médecins Sans Frontières (MSF) was implementing a six-monthly appointment spacing approach adapted locally as *Rendez-vous de Six Mois (R6M)* with an objective to improve retention in care. We sought to evaluate this innovative model of ART delivery in circumstances where access to healthcare is restricted.

**Methods:**

A retrospective cohort study in 2014 of the outcome of a group of stable patients (viral load ≤1000 copies/μl) enrolled voluntarily in R6M compared with a group of stable patients continuing standard one to three monthly visits in Conakry. Log-rank test and Cox proportional hazards model were used to compare rates of attrition (deaths and defaulters) from care between the two groups. A linear regression analysis was used to describe the trend or pattern in the number of clinical visits over time.

**Results:**

Included were 1957 adults of 15 years old and above of whom 1166 (59.6%) were enrolled in the R6M group and 791 (40.4%) in the standard care group. The proportion remaining in care at 18 months and beyond was 90% in the R6M group; significantly higher than the 75% observed in the control group (*p* < 0.0001). After adjusting for duration on ART and tuberculosis co-infection as covariates, the R6M strategy was associated with a 60% reduction in the rate of attrition from care compared with standard care (adjusted Hazard Ratio = 0.40, 95%CI: 0.27–0.59, *p* < 0.001). There was a negative secular trend in the number of monthly clinical visits for 24 months as the predicted caseload reduced on average by just below 50 visits per month (β = −48.6, R^2^ = 0.82, *p* < 0.0001).

**Conclusion:**

R6M was likely to reduce staff workload and to mitigate attrition from ART care for stable patients in Conakry despite restricted access to healthcare caused by the devastating EVD on the health system in Guinea. R6M could be rolled out as the model of care for stable patients where and when feasible as a strategy likely to improve retention in HIV care.

**Electronic supplementary material:**

The online version of this article (10.1186/s12879-017-2826-6) contains supplementary material, which is available to authorized users.

## Background

In 2016, an estimated 19.5 million people living with Human Immunodeficiency Virus (HIV) were receiving antiretroviral therapy (ART) globally. This number is likely to increase exponentially in accordance with the current guidelines of the World Health Organisation (WHO) based on the 2030 vision to end the epidemic of Acquired Immunodeficiency Syndrome (AIDS) [1–5]. The hopes for ending the AIDS epidemic may depend largely on the world’s ability to provide ART to all HIV-infected patients but these hopes may be dashed if retention of people who have already initiated treatment is not optimal. Yet, HIV programmes especially in resource limited settings are faced with the daunting task of retaining the increasing number of people accessing ART [3, 6]. Retention rates of 79.1%, 75.0% and 61.6% at 6, 12, and 24 months, respectively, have been reported in ART programmes in sub-Saharan Africa [7]. A 12-month retention of 79% was reported in five West African countries by The International Epidemiologic Databases to Evaluate AIDS (IeDEA) Collaboration [8]. For ART programmes to continue to expand while retaining people in care, WHO recommends a range of novel models of service delivery that, could be developed and implemented to respond to particular challenges in particular settings [3].

A number of health facility- and community-supported models of ART delivery had been developed by Médecins Sans Frontières (MSF) in collaboration with local governments in sub-Saharan Africa to support on-going efforts to manage an ever-growing cohort of people on ART. These approaches include: appointment spacing for clinical and drug refill visits in Malawi, peer educator-led ART refill groups in South Africa, community ART distribution points in Democratic Republic of Congo (DRC) and patient-led community ART groups in Mozambique. These models have achieved high retention rates ranging from 89% at 12 months in DRC, 92% at 48 months in Mozambique, 94% at 36 months in Malawi, to 97% at 40 months in South Africa [9]. Other countries, including Uganda, South Africa and Zimbabwe, have taken a similar approach that allows for longer supplies of antiretroviral drugs in combination with spaced appointments [9, 10]. These initiatives have so far been applied on stable patients in stable settings only.

In unstable or disaster settings however, the challenges of delivering routine healthcare are even more daunting as efforts are geared towards responding to the disruption caused by a given humanitarian emergency of concern [11]. Before the Ebola epidemic in Guinea, retention on ART at 12-months was approximately 75% [12]. When the outbreak of the Ebola virus disease (EVD) was declared in Guinea in 2014, a significant decrease in health service utilisation for HIV care was reported with rates of defaulting in ART care reaching 42% at the peak of the epidemic [13–15]. Prior to the EVD outbreak, MSF had begun piloting a six-monthly appointment for clinic and drug refill adapted locally as *Rendez-vous de Six Mois (R6M)* for stable HIV patients receiving ART, as a decongestion scheme to relieve pressure on its overstretched referral centre of Matam in Conakry and to improve retention in care. During the Ebola epidemic, the strategy was further deployed with an additional objective to reduce the risk of contracting EVD by reducing frequent contacts with the healthcare facility. We aimed to report this approach implemented during the EVD outbreak when access to healthcare was restricted.

## Methods

A retrospective cohort study to compare attrition from R6M care relative to standard care after a follow up period of 24 months.

### Setting

The HIV epidemic in Guinea remains largely overlooked by the rest of the world due to its low overall prevalence of 1.6%, with approximately only one quarter of people living with HIV (approximately 120,000 in 2014) accessing antiretroviral treatment [12, 16]. MSF has been providing HIV and tuberculosis (TB) services since the start of ART in 2003. In collaboration with the Ministry of Health, MSF provided support to over 7639 HIV patients in 2016 (24% of the national ART cohort) through a decentralised approach in six health centres across the capital city, as well as at an outpatient clinic in Matam district [17]. In 2014, MSF was at the forefront of the Ebola response in West Africa where MSF teams treated 3804 patients in Guinea [18]. The Ebola outbreak had strongly affected Guinea’s ability to provide HIV/AIDS services resulting to poor programmatic outcomes [19, 20].

### The R6M model of care

R6M is a six-monthly appointment (SMA) model of ART care delivery for stable patients developed by MSF and implemented initially by The Chiradzulu HIV program in rural Malawi in 2008 with the objective to reduce patient waiting time and heath staff workload. Stable adults (>95% adherence, current CD4 ≥ 300 cells/μl), not pregnant, on first-line ART for more than a year and not presenting drug intolerance, tuberculosis or Kaposi’s Sarcoma, were scheduled for clinical 6-monthly appointments by nurses and every 3 months for drug refill instead of every 1–2 months for patients in regular ART care [9, 21].

In 2013, MSF began piloting R6M in Conakry with a similar objective and approach to that in Malawi but by using a viral load ≤ 1000 copies/μl (instead of CD4 counts) as the main criterion to define a stable patient because routine viral load monitoring was implemented in MSF supported centres in Guinea. Approximately, 72.5% of the 7250 patients on ART for at least 6 months had had a least one viral load assay in MSF supported centres in June 2016. Of these, 90.7% had achieved viral load suppression. Initial results from implementing R6M prior to the EVD were encouraging [17]. Following the outbreak of EVD in 2014, R6M was scaled up with an additional dual objective of improving retention on ART and reducing the risk of EVD transmission. Patients outside the capital city were provided with ARV drugs enough to cover a period of 6 months while those residing in Conakry were provided with ARVs for 3 months by pharmacy attendants (pharmacy-only visits) and were seen by clinicians every 6 months. Exceptions to the rule were those who developed acute problems that compelled them to return earlier to seek medical attention.

### Study design

A retrospective cohort study of the outcome of a self-selected group of stable patients enrolled into the R6M care compared with a group of stable patients continuing standard care (the control group) at The Matam outpatient clinic supported by MSF in Conakry. Included in the R6M group were patients aged 15 years and above with a current viral load ≤1000 copies/μl, non-pregnant and with no opportunistic infection (OI) between the 1st January 2014 and 31st December 2014. Included in the control group were patients of age 15 years and above with viral load ≤1000 copies/μl, non-pregnant and with no current OI but continuing the routine 1–3 monthly follow-up visits during the same period. All patients eligible for this retrospective records review were included in the study. The choice to be included in either group was voluntary after adequate information had been provided by clinicians as in normal practice. Participants were free to quit or change groups at any time with support from their clinicians. Other baseline characteristics of the study groups were collected for comparability. Patients who were transferred out were excluded from the study irrespective of the group. The date of the last ART viral load defining eligibility was considered as the entry date into the cohort (time 0). The two groups were then observed throughout the duration of the EVD outbreak until 31st December 2015 (time of censoring) to determine their outcomes.

### Data collection

We used routinely collected programme data that had been entered into the TIER.Net® 1.9.2.3 electronic medical record software developed by the University of Cape Town in South Africa. Abstracted for the purpose of the study where sociodemographic variables related to gender and age, clinical data pertaining to incident tuberculosis; current viral load titres and CD4 counts and their dates of measurements; date of ART initiation, current and prior ART regimens; the number of clinical visits; and their outcomes including the dates of ascertainment or censoring of the outcome. The dataset (Additional file 1) was then exported to Stata® software for statistical analysis.

### Statistical analysis

Data analyses were performed using Stata® 14.2(StataCorp LP, TX77845, USA). The data set was checked for logical inconsistencies, illegal codes, omissions and improbabilities by tabulating, summarising, describing and plotting variables. Missing observations were excluded because they constituted a small random proportion.

Our main outcome of interest was attrition from care defined as a composite measure of the rate of occurrence of all-cause deaths and losses to follow-up (LTFU). A patient was classified as LTFU if there was no contact for 90 days or more after the last missed appointment for ARV refill [6]. MSF has a large team of lay workers who were used to provide continuous psychosocial support to patients in care and they were able through monthly home visits and/or telephone calls, to remain in contact with them. This effort in tracing patients was useful to encourage retention in care and to ascertain their outcome. Retention in care was used to indicate the proportion of patients alive and known to be still receiving ART at the time of the study [7]. The rate of attrition as a time to event variable was measured as the number of deaths and LTFU expressed over the length of time in person-months. The number of clinical visits per month was a secondary outcome automatically generated by the data entry software TIER.Net®.

The main explanatory variable of interest was the exposure to R6M or standard model of care following results of the last ART viral load measurement. Other putative variables examined for comparability between the two groups included: gender, age, viral load titre, type of and duration on ART, last CD4 count and occurrence of a clinical event.

Summary statistics were presented as proportions for categorical variables and as means [with standard deviations (SD)] for normal continuous variables or medians [with Interquartile Ranges (IQR)] for skewed continuous variables. Pearson chi-squared tests or Fisher exact tests for small samples were used where appropriate to assess for differences among categorical variables between the two groups. The student t-test was used to test for the mean difference between the groups for continuous variables. Kaplan-Meier survival curves were used to display the rate of attrition over the study period meanwhile the log-rank test was used to assess for differences in survival probabilities between the two groups. A linear regression analysis was used to describe the trend or pattern in the number of clinical visits over time. A univariable Cox regression model was set up to screen for factors associated with attrition. Crude hazard ratios (HR) and their 95%CI were obtained. The *p*-values for hypotheses testing were calculated from likelihood ratio tests (LRT). Variables found to be associated at 5% confidence level, with attrition were included in a multivariable Cox model. Backwards elimination based on *p*-value lower than 0.05 was used to retain variables independently associated with attrition. The survival curves were then adjusted according to these independent predictors of attrition. The corresponding adjusted hazard ratios (aHR), their 95% confidence intervals and *p*-values in the final model were reported [22]. The proportionality hazard assumption over time was assessed graphically using Aalen plots. An “intention- to- treat principle” was applied given that some patients in the R6M group returned to standard care and vice versa [23].

## Results

### Descriptive characteristics of the study groups

**Fig. 1:**
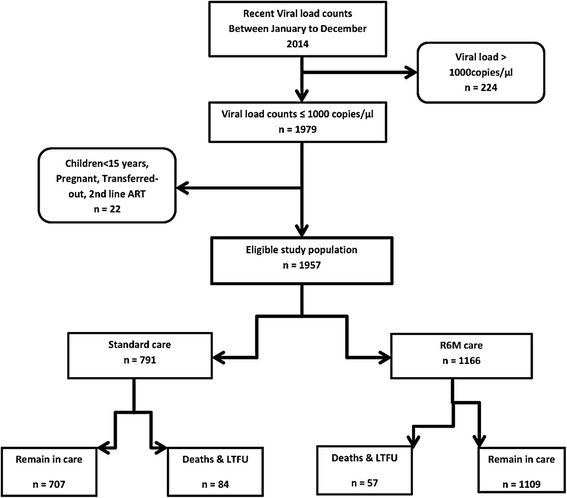
Cohort flow chart

Included in a staggered manner between January and December 2014 were 1957 eligible adults of whom 1166 (59.6%) were in the R6M group and 791 (40.4%) in the standard care group (Fig. 1). During censorship, 202 (17%) participants in the R6M had returned to standard care while 230 (29%) of those in the control group had moved onto the R6M group. The mean period at risk was 16 months per subject or a total analysis time at risk of 31,220 person-months.

Participants in the R6M group were on average about 2 years older than the counterparts in the control group (41.8 ± 10.8 vs. 40.1 ± 10.9 years, *p* = 0.0007). The R6M group had significantly greater proportions of participants currently on Tenofovir (TDF)/Lamivudine (3TC)/Efavirenz (EFV) regimen (82.7% vs. 53.7%, *p* < 0.001), with ART experience of at least 5 years (52.2% vs. 46.1%, *p* = 0.005); and with an undetectable viral load below 250 copies/μl (98.2% vs. 93.7%, p < 0.001) but all had achieved viral load suppression at ≤1000 copies/ μl to become eligible for the study. The two groups were comparable in terms of gender composition, risk of a new opportunistic infection (active tuberculosis) or levels of current follow-up CD4 counts (Table 1).

**Table 1:** Baseline characteristics of the study groups

Characteristics		Standard care group (*n* = 791)	R6M group (*n* = 1166)	*p*-value for difference between the group
Age, mean (SD) years		40.1 (10.9)	41.8 (10.8)	0.0007
Gender, n (%)				
	Male	229 (28.9)	383 (32.8)	
	Female	562 (71.0)	783 (67.2)	
	Total	791 (100)	1166 (100)	0.068
Current CD4 count, mean (SD) cells/μl		497 (261)	494 (243)	0.769
Baseline Viral Load for eligibility in copies/μl, *n* (%)				
	< 250	741 (93.7)	1145 (98.2)	
	250–1000	50 (6.3)	21 (1.8)	
	Total	791 (100)	1166 (100)	< 0.001
TB status at any follow-up visit, *n* (%)				
	Negative/Unknown	778 (98.4)	1150 (98.6)	
	Positive	13 (1.6)	16 (1.4)	
	Total	791 (100)	1166 (100)	0.626
Duration on ART in months, *n* (%)				
	< 60	419 (53.9)	557 (47.8)	
	≥ 60	358 (46.1)	609 (52.2)	
	Total	777 (100)	1166 (100)	0.008
TDF-3TC-EFV regimen, *n* (%)				
	No	366 (46.3)	202 (17.3)	
	Yes	425 (53.7)	963 (82.7)	
	Total	791 (100)	1165 (100)	<0.001
Outcome, *n* (%)				
	In care	707 (89.4)	1109 (95.1)	
	Lost to Follow-up	74 (9.4)	45 (3.9)	
	Dead	10 (1.3)	12 (1.0)	
	Total	791 (100)	1166 (100)	< 0.001

### Trends in attrition from care

**Fig. 2:**
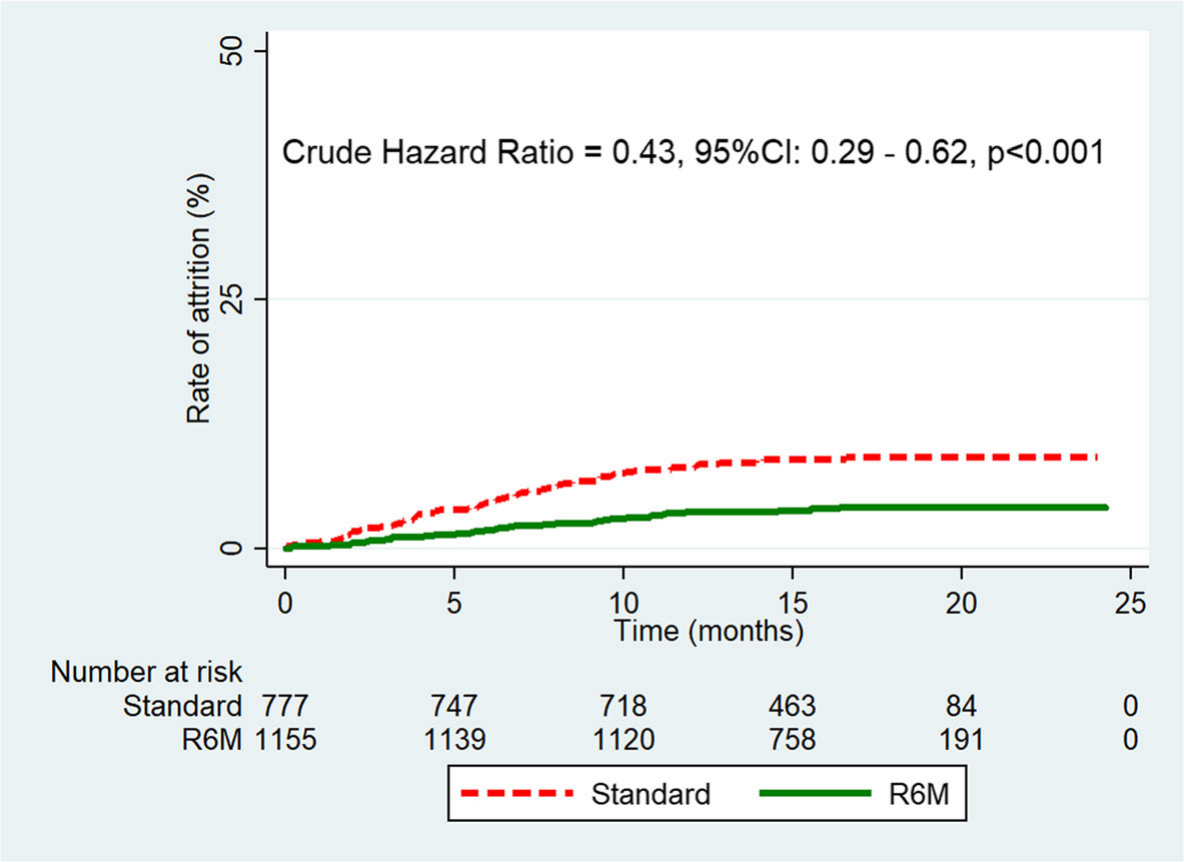
Kaplan Meier curve of attrition from care

After a total observation period of 24 months, 116 participants had dropped out from care through death or LTFU of whom 57 (4.9%) in the R6M group and 84 (10.6%) in the control group (*p* < 0.001). The overall retention rates remained high during the EVD outbreak but were higher in the R6M group than in the control group (Fig. 2): 98.2%, 96.3% and 95.8% at 6, 12 and 18 months respectively in the R6M group against 95.4%, 91.9% and 90.8% at 6, 12 and 18 months respectively in the control group (*p* < 0.0001).

Attrition rates also differed between subjects who became coinfected with tuberculosis during follow-up (27.6% vs: 6.9%) and those who did not (*p* < 0.001). Similarly, persons with an ART experience of 5 years and above had a lower rate of attrition (4.0% vs.10.4%) than those on ART for less than 5 years (*p* < 0.001) as indicated in Table 2. Gender, age, current CD4 counts or viral loads were not associated with the outcome.

**Table 2:** Cox multiple regression model of factors associated with attrition from care (*n* = 1928)

Factor		Proportion of attrition (%)	HR (95%CI)	*p*-value	aHR (95%CI)	*p*-value
Strategy						
	Standard	10.6	1		1	
	R6M	4.9	0.43 (0.29–0.62)	< 0.001	0.40 (0.27–0.59)	< 0.001
Incident tuberculosis						
	No	6.9	1		1	
	Yes	27.6	5.25 (2.56–10.77)	< 0.001	4.35 (2.10–9.01)	0.005
Duration on ART (months)						
	< 60	10.4	1		1	
	≥ 60	4.0	0.42 (0.28–0.62)	< 0.001	0.47 (0.31–0.71)	< 0.001

**Fig. 3:**
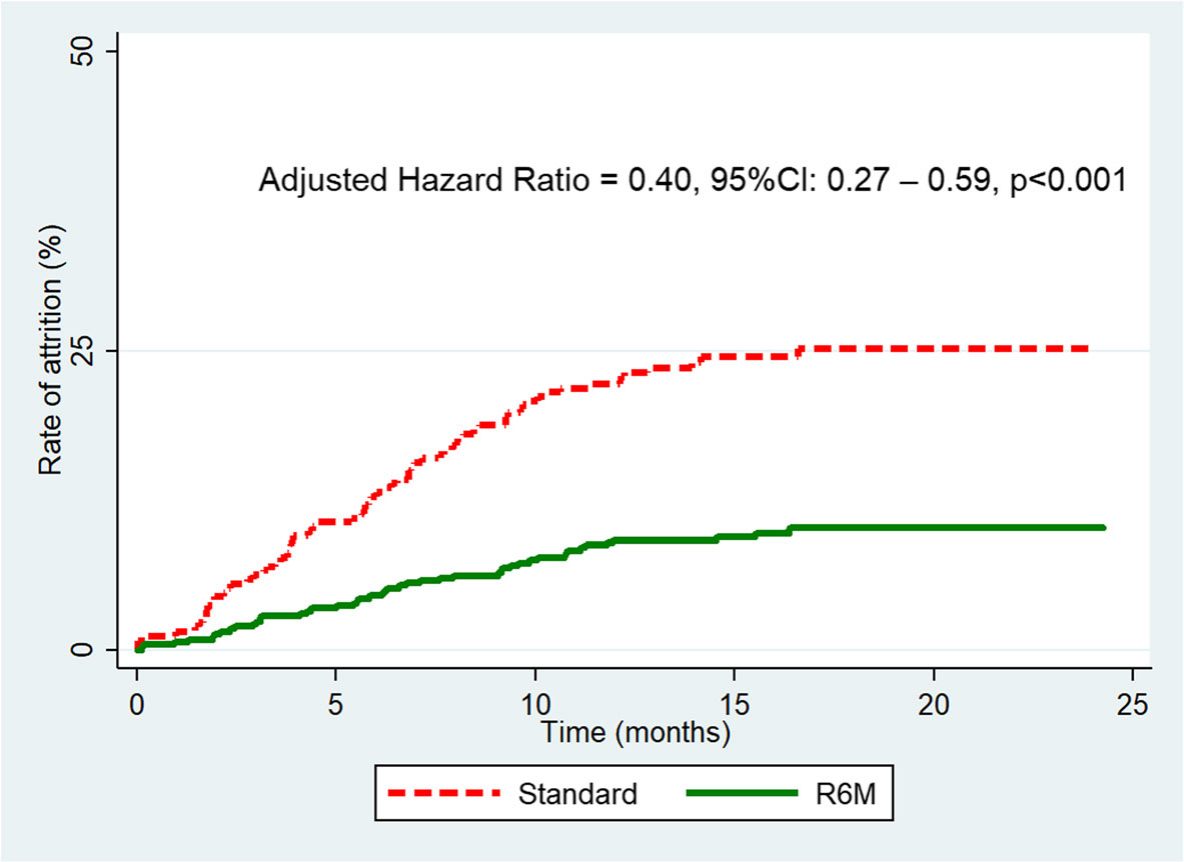
Kaplan Meier curve of attrition in care adjusted for TB coinfection and treatment effect

After adjusting for HIV/TB coinfection and the length of time on ART (Table 2), the proportion remaining in care at 18 months and beyond was 90% in the R6M group (Fig. 3) significantly higher than the 75% observed in the standard group (*p* < 0.0001). R6M was thus associated with a 60% reduction in the rate of attrition from care compared with standard care (adjusted Hazard Ratio = 0.40, 95%CI: 0.27–0.59, *p* < 0.001).

### Trend in caseload over time

**Fig. 4:**
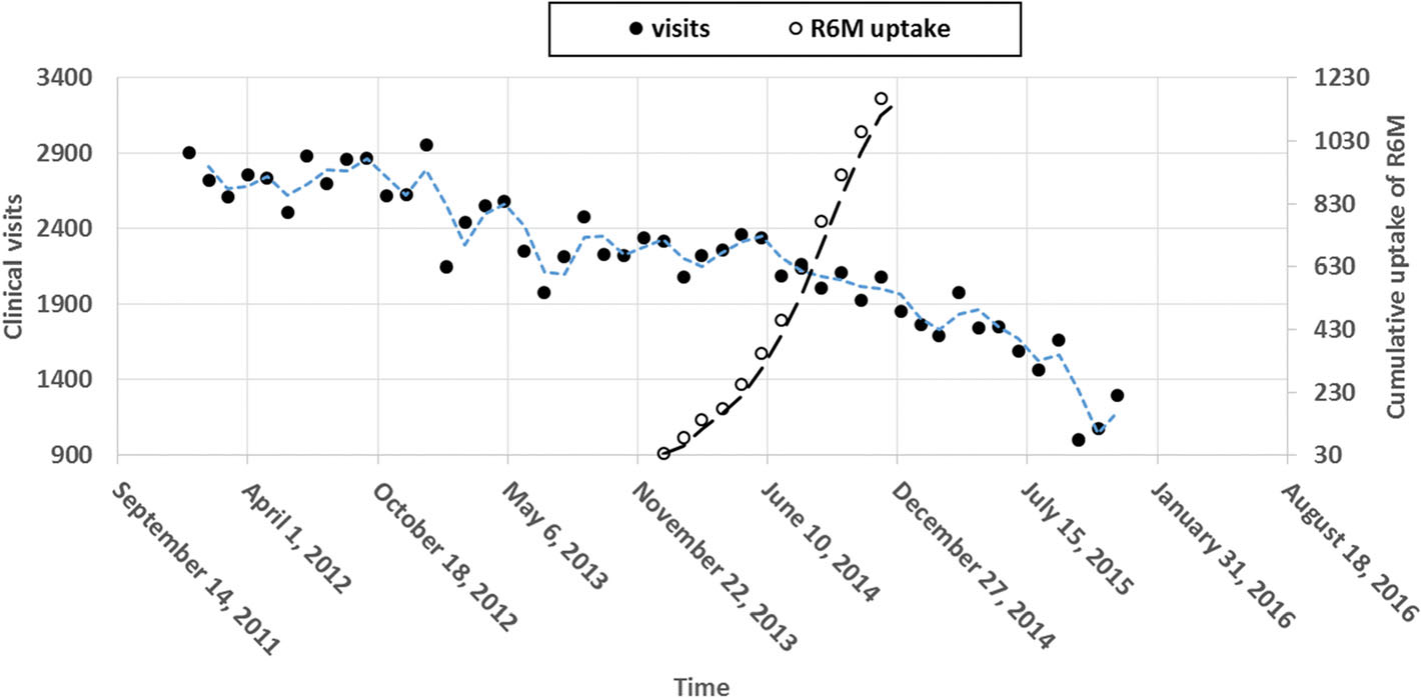
Pattern of clinical visits and uptake of R6M over time

**Fig. 5:**
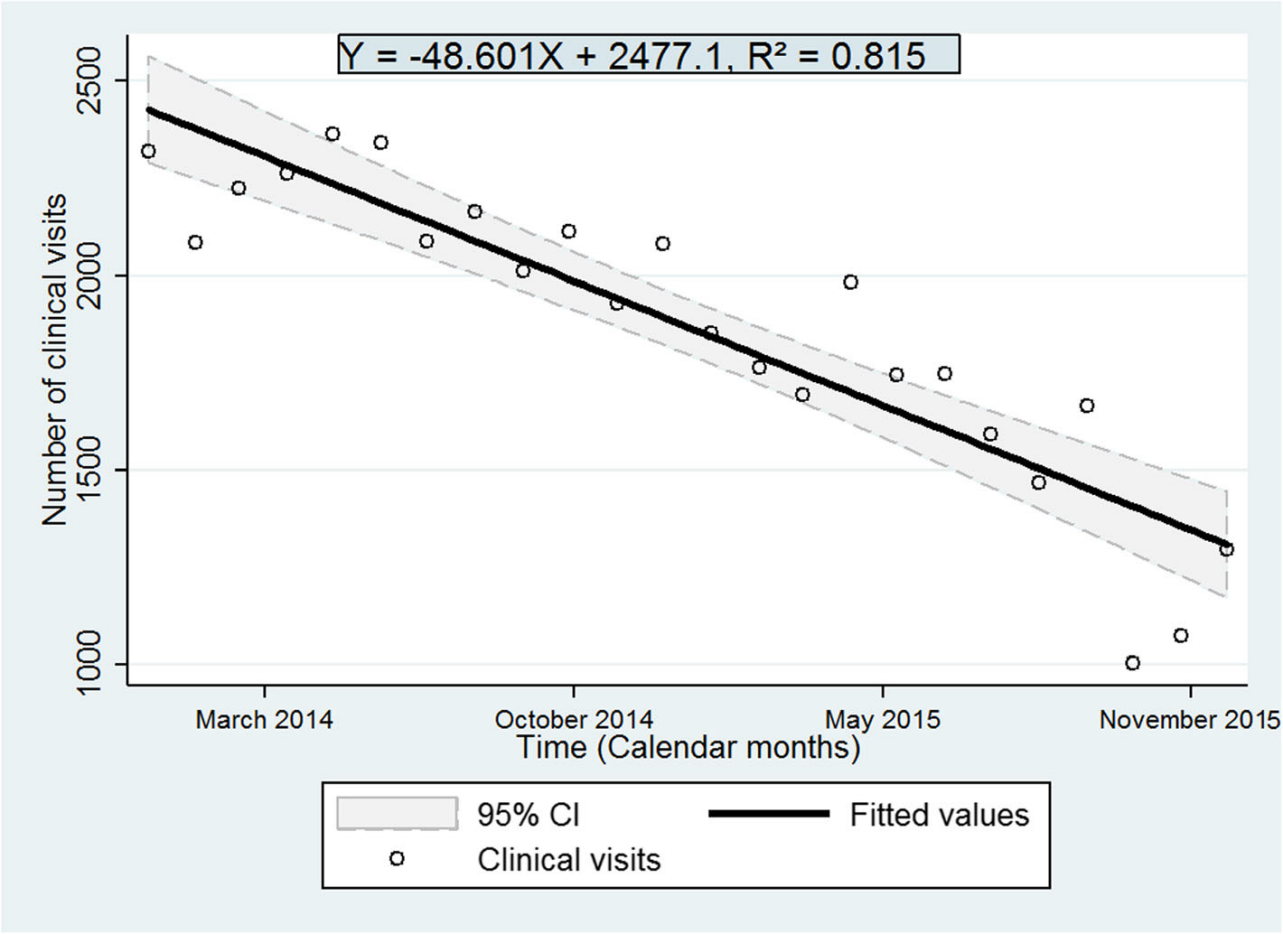
Secular trend and prediction of caseload over time

Before the scale up of R6M in 2014, the number of clinical visits depicted a more or less cyclical or irregular pattern but thereafter, there was a negative secular trend. The number of clinical visits from January 2014 progressively decreased to about 50% by the end of December 2015 at the outpatient clinic of Matam when the uptake of R6M was about 55% of the patients enrolled on ART (Fig. 4). A linear regression model fitted on data from 2014 (Fig. 5) predicted that the number of clinic visits decreased on average by just below 50 every month during this period (β = −48.6, R^2^ = 0.82, *p* < 0.0001). Thus, in practice, R6M as it was implemented, the caseload of clinicians could be estimated according to the following expression:

1 $$ \mathbf{Caseload}=\mathbf{2477}\hbox{--} \mathbf{49}\ \mathbf{visits}\ \mathbf{per}\ \mathbf{month} $$

## Discussion

In this observational study, we compared the R6M approach relative to standard care on the outcomes of stable patients receiving ART during an Ebola epidemic that considerably reduced utilisation of HIV services in Guinea. The study indicated that R6M was likely to reduce attrition in care by 60% for patients who self-selected into the model. We also found that implementation of R6M was linked to a reduction in caseload by about half over a 24-month period at a rate of 50 clinical visits per month on average.

R6M is one of the several strategies for optimising long-term ART delivery developed and implemented by MSF in collaboration with Ministries of Health in sub-Saharan Africa. These models of care have already been integrated into WHO and UNAIDS guidelines and are gaining grounds in a growing number of countries across the continent in flexible ways according to context as “no one strategy fits all” [1, 9, 24]. None of these models has yet been tested in disaster settings. The context in Guinea was and remained one with a low HIV prevalence and a low ART coverage compounded by a devastating EVD that led to massive dropouts in care [12–14, 17]. Despite the disruption in healthcare delivery, stable patients receiving ART in general and those in R6M care in particular, demonstrated a tougher resilience by holding strong to their treatment. The success of a six-monthly appointment (SMA) on retention in care has been described in other settings at different time periods of follow-up. Findings from the Malawian cohort of Chiradzulu district that implemented a health facility driven model like R6M in Guinea had shown a retention rate of 96% at 24 months in 2014 [9]. In community driven models, SMA has equally demonstrated high retention rates: 89% at 12 months follow-up in DRC in 2012, 95.1% after 20 months in rural Mozambique and 95.5% in urban Mozambique in 2013; 92% at 15 months in the Thyolo model of Malawi and 100% at 5 months in the Roma model of Lesotho [24]. In contrast to R6M, and with the exception of DRC that has a low ART coverage (a characteristic common to West and Central Africa); results from studies listed above have been obtained from within Southern African countries with relatively stable and robust health systems to fight against HIV as indicated by their high ART coverage [17, 24, 25]. More so, their results were more descriptive than analytic from a methodological stand point. Results from our study have been derived from a comparative analysis in a disaster context where the HIV epidemic has been largely neglected [12, 17]. Despite differences in settings, uptake levels, implementation models and study designs, the evidences so far have been strong, coherent and consistent in favour of R6M or SMA as a strategy to improve retention in care. Therefore, ART programmes should be encouraged and supported to pick up or roll out to scale the implementation of SMA or any form of long appointment spacing and fast-track drug refill strategy. Some MSF-supported sites with access to routine viral load monitoring such as Malawi, South Africa and Zimbabwe are already moving to a once a year clinical visit with two to three monthly drug refills in between [24].

The National ART Programme in Guinea has endorsed R6M in principle but will have to meet a set of minimum requirements and critical enablers in order to overcome the challenges inherent to its implementation: A sufficient and flexible drug supply chain to permit ART dispensing of up to a 6 months’ supply; a routine viral load testing not only to determine viral load suppression for R6M eligibility but also to detect treatment failure early enough so that patients could return to conventional care before dropping out of care like those who developed active TB in this study; a health information system to tract and monitor patients as they integrate R6M or return to regular care and vice versa since the decision to participate is entirely voluntary and reversible at any time. The programme nonetheless has the potential to face these challenges: the state has been increasing its financial contribution to fight against HIV from 4% in 2011 to about 22% in 2014 [12, 26]; MSF and Solthis (Solidarité thérapeutique et initiatives contre le sida) via the Open Polyvalent Platforms (OPP-ERA) project funded by UNITAID are currently supporting the programme to ensure routine viral load monitoring of treatment response and in the implementation of monitoring and evaluation (M&E) tools based on the electronic health information system. Yet, clinical and immunological monitoring of treatment response as well as paper-based M&E tools already in use can be adapted to fit the R6M model. In rural Malawi for example, a CD4 ≥ 300 cells/μl in the absence of viral load was used as an eligibility criterion [9]. However, immunological and clinical criteria have poor sensitivity and specificity to detect treatment failure, particularly at higher CD4 cell counts [27]. The global response to the EVD in Guinea has also come with some investment for the country to rebuild a stronger health system for all programmes including the ART programme.

A reduction in the burden for healthcare workers and for patients has been the main goal for implementing R6M and community ART groups (CAG). This study has indicated that there had been a steady drop in the caseload for healthcare workers since instituting R6M at the Matam outpatient clinic. Though intuitive to link this reduction in workload to R6M, it is likely that the Ebola epidemic per se did contribute to a reduction in the frequency of contacts with the health facility as well as the decentralisation process integrated with R6M. Despite these intervening circumstances, we strongly believe that R6M can be singled out as the major contributor to the relief of the burden because the negative secular trend did not falter even when the epidemic had substantially receded in late 2015 and because efforts to decentralise patients have been less fruitful as patients do not voluntary accept to abandon the high quality of care offered at this clinic. We did not assess patient or healthcare worker satisfaction or benefits in this study, but given the experience from elsewhere, we can deduce that a reduction in the number of clinical visits would inevitably lead to a reduction in waiting times, travel costs and stigma; to improvement in individual patient empowerment to self-management, and of course to improvement in retention in care from the patient perspective [9, 24, 28]. Despite these benefits, some patients preferred to remain in conventional care because they perceived regular professional contact as more reassuring. From the healthcare service perspective, a reduction in the number of clinical visits would equally mean a reduction in the cost of healthcare delivery, a likely reduction in the risk of burn out syndrome and an improvement in the quality of care [29, 30]. An economic evaluation as well as a patient/provider satisfaction surveys are recommended to validate these assumptions and to measure their real effects.

The study had other limitations. In routine care, patients “self-selected” conveniently or voluntarily into either treatment option. This obviously introduced selection bias but we thought that by choosing a ‘control group’ made of “stable” patients who were also eligible for R6M but opted to remain in conventional care after receiving adequate information including those related to knowledge and attitudes, from their clinicians, this could somehow reduce the bias. Collecting additional baseline data from both groups was done to identify and then control for any known differences between the two groups. Protocol violations were common because we observed that 17% of participants in the R6M group returned to conventional care while 23% of those in regular care opted for R6M during follow-up. This is a true reflection of routine care and of an observational study with a likely implication being a null effect of R6M but we believe this was taken care of by the “intent-to-treat” approach in the analysis. The period of observation of 24 months whereby only a maximum of four visits (against up to 24 visits for those in the control group) were possible for participants in the R6M group was rather short for a sufficient number of events to occur in this group. However, the R6M group was large enough to allow for accumulation of events in the group. Lack of randomisation in the design meant causality could not be assumed. The study did not directly measure the effect of R6M on the transmission of EVD but we can equally assume that if the number of clinician-patient contacts was reduced coupled with a reduction in waiting times and congestion in waiting rooms, the probability of an effective contact between a potential EVD contact or victim and a healthy person was also reduced [31]. Mathematical modelling could be recommended to simulate disease transmission dynamics in such a context.

## Conclusions

R6M was likely to reduce staff workload and to mitigate attrition from care for stable patients in Conakry despite restricted access to healthcare caused by the devastating EVD on the health system in Guinea. R6M could be rolled out as the model of care for stable patients where and when feasible as a strategy to improve retention in HIV care.

## Additional file

Additional file 1: The dataset (Additional file 1) was then exported to Stata® software for statistical analysis. (XLSX 199 kb)

## Abbreviations

3TC: lamivudine or 3-thiacytidine
aHR: adjusted Hazard Ratio
AIDS: Acquired Immunodeficiency Syndrome
ART: Antiretroviral Therapy
ARV: Antiretroviral (drug)
CAG: community ART groups
CD4: Cluster designation 4 (of lymphocytes)
EFV: Efavirenz
EVD: Ebola Virus Disease
HIV: Human Immunodeficiency Virus
HR: Hazard Ratio
IeDEA: The International Epidemiologic Databases to Evaluate AIDS
IQR: Interquartile range
LTFU: Loss to follow up
MSF: Médecins Sans Frontières
OI: Opportunistic infection
R6M: *Rendez-vous de Six Mois (R6M)*
SD: Standard deviation
SMA: Six-monthly Appointment
TB: Tuberculosis
TDF: Tenofovir disoproxil fumarate
WHO: World Health Organisation
